# Recurrent Mass Hysteria in a Secondary School in Nepal: A Case Report

**DOI:** 10.31729/jnma.v63i290.9205

**Published:** 2025-09-01

**Authors:** Samata Nepal, Anu Marhatta, Alok Atreya, Ritesh Menezes, Sajja Shrestha, Shikha Pahari

**Affiliations:** 1 Lumbini Medical College, Palpa, Nepal; 2 College of Medicine, Imam Abdulrahman Bin Faisal University, Dammam, Saudi Arabia

**Keywords:** *health education*, *mass hyMeria*, *psychogenic illness*, *secondary school*, *Nepal*

## Abstract

Mass hysteria is a rare phenomenon that can occur in any community. We report a case of recurrent mass hysteria in a co-education secondary school in Palpa, Nepal. The index case was a 16-year-old girl who exhibited abnormal behavior and was diagnosed with psychotic depression. After a few days, several other girls in her class exhibited similar symptoms, culminating in a mass conversion disorder in the school assembly. The situation caused widespread panic among students, teachers, and staff members, ultimately leading to the school’s closure. Health workers from the regional tertiary healthcare institute intervened by providing health education to parents, guardians, and students, which was the primary interventional strategy employed. In this case report, we describe the case, discuss the significance and implications, and emphasize the importance of increasing awareness and early diagnosis and treatment of such conditions.

## INTRODUCTION

Conversion disorder, also called functional neurological symptom disorder, maybe triggered by distress, past trauma, or stressor.^1,2^ International Classification of Diseases (ICD)-10 classifies it as a motor type of dissociative disorder, whereas Diagnostic and Statistical Manual of Mental Disorders (DSM)- 5 categorizes it under somatoform disorders.^2-4^ Its symptoms include loss of consciousness, twisting of limbs, pseudoseizure, breathing difficulty, shouting, irrelevant talking with no underlying medical explanations.^1,5,6^ Mass hysteria or mass psychogenic illness (MPI), is a conversion disorder outbreaks in a cohesive group, without physical, biological, or etiological causes.^7^

Data on the global MPI burden are limited. However, studies in Nepal show that the outbreak occurs in rural schools/ communities, causing school closure.^8-11^

## CASE REPORT

The case report is about an epidemic of abnormal behavior that occurred in a co-educational secondary school in Palpa, Nepal.

The first case occurred a few days before mass hysteria in a 16-year-old girl in grade 9, who exhibited abnormal behavior, such as tremors, shouting, screaming, and episodic loss of awareness while the teacher was teaching. She was then taken to the teacher’s room in an unconscious state, and her mother was informed. She regained consciousness soon after, but she denied having any recall of the incident. When her mother arrived, she accused a witch of taking over her daughter. She was then taken to faith healers to relieve her from the witch’s spell. The girl continued to show such bizarre symptoms like shouting, body tremors, and irrelevant talk. After persuasion from teachers and neighbors, she was then taken to a medical facility, where she was diagnosed with psychotic depression and prescribed olanzapine 2.5 mg and sertraline 25 mg daily for a month. However, after a few days off the medication, the symptoms resurfaced. Several girls from her class, also exhibited similar episodes of disorganized behavior, and soon after, several students showed equivalent abnormal behavior.

During a morning assembly, when the principal addressed the situation, a few students started showing abnormal behavior, and it spread among the students. One after another, 31 female students were affected on 30^th^ November 2022, and many more episodes were witnessed for several days and even months. Despite clinical differences observed in students affected by mass conversion, those who witnessed it, victims, and other students believed it was caused by an “evil spirit.” Some teachers speculated that it was due to sexual desire in teenage girls resulting from hormonal changes.

The principal of the school sought help from Lumbini Medical College Teaching Hospital (LMCTH), the tertiary health care institute of that region. The first author and the team at LMCTH were called upon to investigate and manage the epidemic. With the telephone consultation from LMCTH and prior knowledge, the situation was managed by the teachers and staff members who separated the students into different rooms and contacted their parents and guardians. An on-the-spot visit was made by a team of health workers from LMCTH, who interviewed most of the teachers, the principal, the administration of the school, parents, caregivers, and some students exhibiting peculiar symptoms.

Interventions were made by providing health education to the parents, guardians, and students about the stigma of mental health and the importance of seeking medical treatment for medical illnesses rather than relying on traditional methods or faith healers. This was done by delivering a series of lectures by the faculty members of LMCTH. The focus was on delivering awareness about how to deal with such situations, including the process of segregation and referral for psychological counselling at LMCTH, if needed. The psychiatrist re-evaluated the index case, who was previously diagnosed with psychotic depression.

For a period of 2-3 months, a series of similar attacks occurred, including the index 16-year-old, who received psychiatric treatment at LMCTH. Several similar cases were seen in the emergency department, and sporadic cases were reported among students at the school and neighboring schools. The team kept in contact with the principal for follow-up, but no further mass hysteria has been reported at the school to date.

Following the interventions, the 16-year-old index case faced significant social repercussions. Due to community beliefs that attributed her symptoms to ‘casting witch-spells,’ she was stigmatized and barred from continuing her studies at the school. Her family decided to get her married. This decision was influenced by local perceptions and pressure from parents and community members, despite her ongoing psychiatric treatment at LMCTH.

## DISCUSSION

The epidemic affected 31 female students, with symptoms including disorganized behavior and physical helplessness. Female students seem to have a higher attack rate than male students, suggesting that gender may be a risk factor, especially in adolescents.^12^ Psychological stressors were apparent in the areas of school, family, and community. Traditional beliefs still prevail, even among educated people.

The phenomenon of mass hysteria has been reported in numerous cultural, ethnic, and religious groups throughout the world since ancient times.^13^ Studying mass hysteria is essential because it provides insight into how social and cultural factors impact mental and physical health, as well as the power of suggestion, the role of fear and anxiety in shaping our attitudes and behaviors, and the significant public health implications it can have.^8,14^

Conversion disorders are prevalent and can cause significant morbidity and socioeconomic problems. The primary treatment of the condition is identifying and eliminating psychological stressors. Rapid resolution of symptoms is seen once the individual is removed from the environment where the outbreak occurred.

Mass hysteria can have significant medicolegal implications, particularly in situations where it leads to panic, injury, or loss of life. In the present case, the index patient was stigmatized for casting witch-spells and was barred from continuing her studies. This highlights the profound social and cultural impacts of MPI in Nepal.^8^ Outcomes like these depict the power of fear-driven suggestion in shaping community behaviors and the urgent public health need to address stigma through education.^6,10,15^

It is inhumane to drop out a 16-year-old girl and get her married instead of treating her for the treatable mental illness. The perception that a disease outbreak is caused by a mass psychogenic illness could lead to delayed diagnosis and treatment, potentially leading to further harm.^1,6,7,14,15^ In some cases, individuals who are thought to have been affected by mass hysteria may seek medical treatment and be diagnosed with a psychogenic illness, leading to potential medicolegal implications in terms of disability claims, insurance coverage, and legal liability for medical professionals who may be involved in their care. Unlike other physical illnesses, which heal soon, treatment of mental illness takes time, and there are many barriers to seeking care for mental illness in the context of Nepal.^15^

In conclusion, the issues surrounding conversion disorder, mass hysteria, and their potential medicolegal implications highlight the need for increased research and understanding in these fields. It is essential to approach these conditions with empathy and avoid stigmatizing those who experience unexplained physical symptoms or illnesses. Additionally, the belief in traditional practices and superstitions in some cultures can exacerbate the consequences of mass hysteria, leading to panic, injury, or loss of life. Therefore, it is crucial to educate the public about these conditions and their potential consequences to promote early diagnosis and treatment, minimize harm, and prevent further morbidity and socioeconomic problems.
